# Iron Absorption in *Drosophila melanogaster*

**DOI:** 10.3390/nu5051622

**Published:** 2013-05-17

**Authors:** Konstantinos Mandilaras, Tharse Pathmanathan, Fanis Missirlis

**Affiliations:** 1 School of Biological and Chemical Sciences, Queen Mary, University of London, Mile End Road, London, E1 4NS, UK; E-Mail: k.mandilaras@qmul.ac.uk; 2 Department of Physiology, Biophysics and Neuroscience, CINVESTAV-IPN, IPN Avenue 2508, Zacatenco, 07360, Mexico City, Mexico; E-Mail: dashi05@fisio.cinvestav.mx

**Keywords:** metal, heme, transferrin, Dcytb, micronutrient, immunity, hemolymph, neurodegeneration, spermatogenesis, circadian rhythm

## Abstract

The way in which *Drosophila melanogaster* acquires iron from the diet remains poorly understood despite iron absorption being of vital significance for larval growth. To describe the process of organismal iron absorption, consideration needs to be given to cellular iron import, storage, export and how intestinal epithelial cells sense and respond to iron availability. Here we review studies on the Divalent Metal Transporter-1 homolog Malvolio (iron import), the recent discovery that Multicopper Oxidase-1 has ferroxidase activity (iron export) and the role of ferritin in the process of iron acquisition (iron storage). We also describe what is known about iron regulation in insect cells. We then draw upon knowledge from mammalian iron homeostasis to identify candidate genes in flies. Questions arise from the lack of conservation in *Drosophila* for key mammalian players, such as ferroportin, hepcidin and all the components of the hemochromatosis-related pathway. *Drosophila* and other insects also lack erythropoiesis. Thus, systemic iron regulation is likely to be conveyed by different signaling pathways and tissue requirements. The significance of regulating intestinal iron uptake is inferred from reports linking *Drosophila* developmental, immune, heat-shock and behavioral responses to iron sequestration.

## 1. Introduction

Iron is an indispensible micronutrient for the development of *Drosophila melanogaster* [1,2]. Enzymes that bind iron, heme or iron-sulfur clusters carry out numerous physiological functions, including respiration [3] and the synthesis of DNA [4,5], ecdysone [6,7], dopamine [8] and lipids [9]. Mitochondria are the site of respiration and synthesis of heme and iron-sulfur clusters and respond to the cellular sensing systems for oxygen and iron [10,11,12,13,14]. Despite the elucidation of key biochemical requirements for iron, our knowledge of how iron is acquired from the diet of *Drosophila* larvae or adults and distributed to its various target tissues and proteins in a regulated manner remains at a rudimentary level [15]. Here, following a summary of how iron absorption occurs in mammals [16], we describe early studies of iron homeostasis in *Drosophila* that used histochemical and radioactive methods [17], atomic absorption spectrometry [18] and electron microscopy [19] to detect iron. Then, more recent studies of particular genes involved in iron absorption are described. We also identify key genes that are conserved between *Drosophila* and mammals and are predicted to function in iron absorption. Despite many similarities, some of the players with known roles in mammals are not conserved in *Drosophila*. Therefore, significant gaps remain in our present knowledge of how iron is acquired from the insect’s diet. Yet, the immune response, the maintenance of circadian rhythms and a number of developmental and aging-related processes are known to depend on iron, meaning that further research into iron homeostasis in the *Drosophila* model is required. 

## 2. Brief Overview of Iron Absorption in Mammals

**Figure 1:**
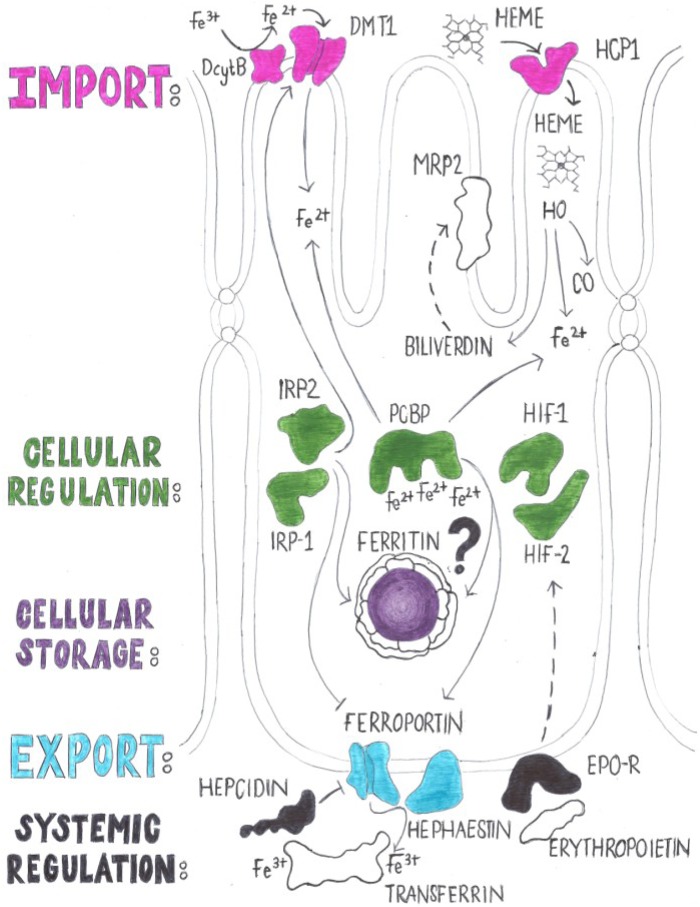
Simplified scheme of iron absorption in mammals. A typical enterocyte of the duodenum of the mammalian intestine has uptake transporters for iron (DMT1) and heme (HCP1) localized in the apical membrane. An iron export transporter (ferroportin) is localized in the basolateral membrane. Ferric iron is reduced by Dcytb prior to import and oxidized by Hephaestin upon export. Iron is stored locally in the enterocyte in ferritin. Whether the iron chaperone PCBP has a role in iron absorption remains to be determined (indicated by a question mark). Heme oxygenases release iron from heme. The large byproduct of this reaction (biliverdin) is modified and secreted into the gut lumen though the Multidrug Resistant Protein-2 (MRP2) transporter. Iron absorption is regulated at the systemic level by hepcidin, which is secreted by the liver hepatocytes in response to various physiologic stimuli. Local cellular regulation also occurs via the Hypoxia Inducible Factors (HIFs) and Iron Regulatory Proteins (IRPs) and may be influenced by circulating levels of erythropoietin (EPO).

A number of comprehensive reviews have been published recently that describe iron absorption in mammals [16,20,21,22,23], so only a very brief summary is presented here as a means to introduce the key proteins involved in the process. First we describe the transport of iron through the epithelial cell in the duodenum [24] and then we discuss the regulation of this process by systemic signals (Figure 1).

### 2.1. Iron Trafficking through the Enterocyte

Iron absorption is complete when the metal ion has crossed the duodenal enterocyte and has been delivered to transferrin in the circulatory system [25]. Divalent metal transporter-1 (DMT1) is currently the only known transporter for the cellular uptake (import) of non-heme iron [26]. The duodenal lumen is an oxidizing environment where most iron is present in the ferric state, yet only the reduced (ferrous) form of iron is transported through DMT1. To facilitate iron absorption, duodenal cytochrome b (Dcytb) reduces ferric iron [27]. Iron is also absorbed in the form of heme, which is internalized through the heme carrier protein-1 (HCP1) [28], which also transports folate [29]. Heme oxygenases breakdown heme into CO, ferrous iron and biliverdin [30]; biliverdin is further converted to bilirubin by Biliverdin Reductase. The Multidrug Resistant Protein-2 (MRP2) is localized in the villi [31] and can export bilirubin from the cell [32]. Currently, the mechanism of iron trafficking through the cell is not fully understood. Specifically, it remains to be determined whether Poly(C) binding proteins (PCBPs), which can bind three ferrous irons and deliver them to ferritin [33], have a function in iron absorption or whether iron traffics in a labile form. Ferritin sequesters iron in the enterocyte and blocks its release to the circulation in a regulated manner [34]. Iron is released from enterocytes by ferroportin [35], from which it is released only after oxidation by the multicopper oxidase (MCO) Hephaestin [36]. The ferric iron is then bound by transferrin, which is the main source of iron for cells in peripheral tissues expressing the transferrin receptor-1 [37]. Copper is also required for iron absorption because it is a prosthetic factor in Hephaestin (reviewed in [38]).

### 2.2. Regulation of Iron Absorption

Iron absorption depends both upon cellular Iron Regulatory Proteins (IRPs) and Hypoxia Inducible Factors (HIFs) and upon systemic regulation through the iron hormone hepcidin [39]. Hepcidin is secreted by liver hepatocytes in response to iron-related stimuli [39]. Hepcidin binds directly to ferroportin on enterocytes and other cells, which promotes the internalization and degradation of the protein [40]. IRPs respond to low iron by repressing the translation of ferritin, ferroportin, mitochondrial aconitase and HIF-2α, while they increase expression of DMT1 and Transferrin Receptor-1 (TfR1) via Iron Responsive Elements (IREs) present in the 5′UTR and 3′UTR, of the respective mRNAs [41,42]. Oxygen levels also have an impact on IRP activation [43]. HIF-2α is a major player in regulating iron absorption by directly controlling the transcription of iron transporters in the intestine [44,45,46,47]. Hypoxia is also sensed in the kidney, which releases erythropoietin (EPO) into circulation [48,49,50]. As erythropoiesis requires high amounts of iron for the production of new red blood cells, it is no surprise that duodenal enterocytes have responsive erythropoietin receptors (EPO-Rs) [48,49,50]. Thus, peripheral tissues can systemically regulate iron absorption by signaling to the intestinal mucosa to absorb iron more actively and in addition the mucosal cells can also actively sense iron and oxygen levels. 

## 3. Early Studies of Iron Homeostasis in *Drosophila*

This review focuses on studies that involved *Drosophila melanogaster*, however it is important to highlight at the outset a number of excellent review articles that describe relevant studies in other insects [51,52,53], including recent publications focusing on insect iron-storage ferritin complexes [54] and high-affinity, iron-binding transferrin proteins [55].

**Figure 2:**
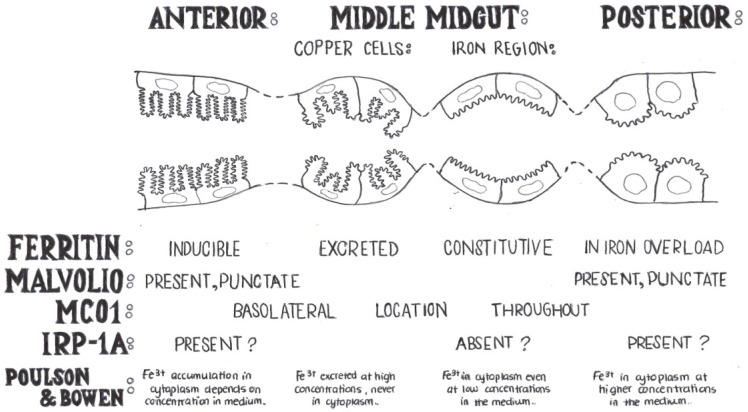
Iron absorption likely takes place in the anterior midgut where all transport proteins studied to date are found. Copper cells are acid-secreting cells, which have also been shown to secrete iron-loaded ferritin in the intestinal lumen. The iron cells express ferritin constitutively and likely serve an iron storage function. Iron cells regulate iron homeostasis independently of IRP-1A and ferritin independently of iron. The posterior midgut appears to be involved with iron homeostasis only in conditions of iron overload. How the different intestinal domains interact with each other remains unknown. For higher resolution images of the different cell types in this diagram the readers are referred to a beautiful representation based on ultrastructure studies performed recently for this tissue [57]. Our diagram is also based on the now 61-year-old study of iron and copper localization in insects [17]. The most posterior part of the midgut (not shown) has no involvement in iron homeostasis, but is a compartment specialized in zinc storage [58,59].

In 1952, Poulson and Bowen presented an elegant demonstration of their histochemical findings on ferric iron and copper present in the intestine of *Drosophila* [17]. These authors used ferrocyanide (Prussian Blue Stain) to visualize iron while modifying the amounts of iron in the diet. In addition, they used radioactive Fe^59 ^to trace the element in the larval tissues. They detected an iron region in the middle midgut and showed that iron added in the diet accumulated in the anterior midgut. Their speculation that iron was present in the form of ferritin was proven correct later [19]. In high doses of dietary iron, ferritin iron was excreted from calycocytes (also known as copper cells) and also made its way to the posterior midgut. Despite significant progress in sixty years, the opening remark by Poulson and Bowen continues to resonate today [17]: “*Although the importance of the inorganic constituents of cells has long been recognized, remarkably little is actually known concerning their specific localizations and functions in the structure and economy of the cell.*” We have modified and redrawn their introductory figure adding new molecular findings to corroborate their classic work (Figure 2).

The next seminal paper presenting a significant advance in the field describes work in another insect, the lepidopteran *Calpodes ethlius*. Larvae were again reared with diets differing in iron concentration and observed under the electron microscope using fixation conditions that permit direct visualization of the endogenous ferritin [19]. Locke and Leung confirmed that iron was stored in ferritin particles that were abundant in the secretory pathway of intestinal cells. This was in contrast to the situation in mammals, where ferritin resides in the cytosol. However, many controls were used to prove this point beyond doubt [19,56]. These authors also reported that ferritin was secreted to the gut lumen in iron overload conditions.

Prior to the era of molecular cloning, Massie *et al*. [18] demonstrated that iron accumulates during the ageing process in *Drosophila*. These authors also suggested that inhibiting iron absorption could enhance the lifespan of fruit flies [60]; however, their statement should be qualified since their experimental set up used dietary tea extracts. Looking at how iron was sensed in cells of *Drosophila*, Rothenberger *et al*. [61] demonstrated that *Drosophila* also carried an active IRP/IRE system. 

## 4. Genes with a Known Function in Iron Absorption in *Drosophila melanogaster*

The first iron-related genes to be cloned in the fly were those encoding the subunits of the iron-storage ferritin protein [62,63], transferrin (Tsf1; [64]) and two IRP1 homologs [65]. Reverse genetic approaches, *i.e.*, the discovery of mutations in genes that caused specific phenotypic alterations led to the characterization of the DMT1 homolog *Malvolio* (*Mvl*; [66]) and of the melanotransferrin homolog *Tsf2* [67]. The *Drosophila* genome sequence [68] informed studies on a mitochondrial form of ferritin [69] and on MCOs [70,71]. Table 1 summarizes the key studies on these genes and this section describes what is known about the role of these proteins in relation to dietary iron absorption.

**Table 1:** Proteins with known functions in *Drosophila* iron absorption.

Protein name (mammals)	Protein name (*Drosophila*)	Key role	References
DMT1	Malvolio	*Malvolio* mutants are iron deficient and show behavioral defects.	[66,70,72,73]
Ferritin	Ferritin	*Drosophila* ferritin is a secreted protein required for iron storage and also iron absorption.	[2,15,54,62,63,74,75,76]
Transferrin	Tsf1	*Tsf1* is an immune-responsive gene. Whether it traffics iron between cells remains unclear.	[53,55,64,77,78]
Melanotransferrin	Tsf2	*Tsf2* is required for the assembly of septate junctions in epithelial cells.	[67]
Hephaestin	MCO1, MCO3	MCO1 and MCO3 are putative ferroxidases. Both show loss-of-function phenotypes with respect to iron homeostasis.	[70,71]
IRP1, IRP2	IRP-1A	IRP-1A regulates ferritin and succinate dehydrogenase translation via IREs.	[61,79,80,81,82,83,84,85,86]

### 4.1. Mvl, the Drosophila Homolog of DMT-1

*Mvl* is the *Drosophila* DMT1 homolog, originally identified in a mutagenesis screen for genes affecting taste behavior in flies [66]. *Mvl* mutants lost a characteristic sugar-preference trait of wild type flies and this phenotype could be rescued by exposure to excess dietary iron [72]. Tissue staining with a specific antibody revealed that the Mvl protein resides in the anterior and posterior parts of the middle intestine, as well as in the Malpighian tubules, brain and testis [73]. Iron stores are depleted in *Mvl* mutants [70,87]. The *Mvl* mutant also suppressed iron accumulation in the intestine, caused by RNA interference (RNAi) of ferritin in this tissue [74]. Hence, sufficient evidence supports the conclusion that Mvl is an iron import protein of *Drosophila*.

### 4.2. Ferritin

*Drosophila* makes two types of ferritin: the testis-specific mitochondrial protein encoded by the X-chromosome *Ferritin-3-Heavy-Chain-Homolog* (*Fer3HCH*) gene, which has no known contribution to iron absorption and a minor (or testis-specific) role in overall iron homeostasis [69], and the major secretory type responsible for systemic iron storage [2,15] and iron absorption [74]. *Drosophila* ferritin is the product of two genes, *Fer1HCH* and *Fer2LCH*, located adjacent to each other on the left arm of the 3rd chromosome [75]. Fer1HCH has ferroxidase activity required for iron loading and Fer2LCH provides the iron nucleation sites required for the mineralization of the ferrihydrite iron core [2,62,63,76]. Each ferritin molecule is composed of 12 Fer1HCH and 12 Fer2LCH subunits [2,74,76]. Radioactive tracing showed that most ingested iron accumulates in *Drosophila* ferritin [2,69]. Analysis of single insertion mutants that disrupt *Fer1HCH* and *Fer2LCH*, respectively, showed that disruption of either gene product reduces total ferritin levels in whole flies and leads to embryonic or early larval lethality [2]. Midgut-specific RNAi of ferritin resulted in iron accumulation in the intestine but systemic iron deficiency [74].

### 4.3. MCOs

*Drosophila melanogaster* has four MCOs in its genome [88]. There is preliminary biochemical evidence that *Drosophila* MCO1 can oxidize ferrous iron; and RNAi of *MCO1* resulted in iron-depleted flies consistent with the proposal that MCO1 is an intestinal ferroxidase implicated in iron absorption [71]. In contrast, *MCO3* mutants accumulated more iron in the iron region of the intestine and restored the depleted iron stores of *Mvl* mutants, but with milder effects in overall iron homeostasis [70]. A comprehensive biochemical characterization of these enzymes is needed to confirm their proposed ferroxidase activities.

### 4.4. Transferrins

Tsf1 is an abundant protein in the hemolymph [64,78]. Whether it traffics iron between tissues remains unclear. Ventral furrow formation during the early development of the *Drosophila* embryo appears to require differential regulation of Tsf1 in ventral and lateral cells, but the mechanistic details of why this needs to be so, are not understood [77]. There is evidence that *Tsf1* is an immune-responsive gene [64,78]. Overall, further studies are required to determine whether *Drosophila* Tsf1 performs a similar role to mammalian transferrin in serving as an iron transport carrier between cells. 

More is known about *Tsf2*, the fly homolog of melanotransferrin, whose function in mammals remains unclear except for its up-regulation in melanomas [89]. In a seminal paper, Tiklová *et al*. [67] showed that septate junction assembly during epithelial maturation relies on endocytosis and apicolateral recycling of *Tsf2*. Tsf2 is a component of the epithelial septate junctions. In particular, the binding of iron to Tsf2 was shown to be required for the epithelial structure that blocks paracellular iron absorption to be formed [67]. A third homolog of transferrin, *Tsf3*, has not been functionally characterized to date [53,55].

### 4.5. IRP/IRE

A protein with IRE binding activity and IREs present within the 5´UTRs of the mRNAs of *succinate dehydrogenase subunit B* and *Fer1HCH* were described soon after the discovery of this regulatory system in mammals [13,65,79,80,81,82,83,84]. The IREs appear to have evolved independently in this lineage by convergent evolution targeting the same key players [85]. The logical implication is that under iron deficiency, ferritin (each molecule of which can bind thousands of iron atoms) and the citric acid cycle (which, coupled to oxidative phosphorylation, accounts for a significant portion of the mitochondrial iron proteins) need to be physiologically suppressed. *Drosophila* makes two highly homologous IRP1-like proteins (IRP-1A and IRP-1B) encoded by different genes [65]. In the evolution of the IRP/IRE system, the ancient cytosolic aconitase was duplicated in insects with one variant (IRP-1A) acquiring IRE-specific binding [86]. Studies of ferritin regulation in the intestine suggested that IRP-1A is absent from the iron region, but that it regulates ferritin in the anterior and posterior midgut (Figure 2) [15,90].

## 5. Genes with a Known Function in Iron Absorption in Mammals that Are Conserved but Have Not Been Studied in *Drosophila melanogaster*

In this section we consider other key players in mammalian iron absorption (Figure 1) that are conserved in *Drosophila*, but where studies are lacking either entirely or with respect to the function of these genes in iron absorption (Table 2).

**Table 2:** Genes with putative functions in *Drosophila* iron absorption.

Protein name (mammals)	Putative homologous genes in *Drosophila*	Comments	References
Dcytb	*CG1275*; *nemy*	One of two fly homologs (nemy) has a function in learning and memory.	[91,92]
HCP1	*CG30345*	Flybase reports low levels of expression for this gene possibly involved in cellular heme uptake.	[93]
FLVCR	*CG1358*	Flybase reports low levels of expression for this gene possibly involved in cellular heme export. RNAi in clock neurons caused disrupted circadian rhythms.	[93,94]
HO1, HO2	*HO*	HO is required for development; it degrades but is not inducible by heme.	[95,96]
HIFα, HIFβ	*sima*; *tango*	HIF signaling is conserved in *Drosophila* but not studied in the context of iron.	[97,98,99,100,101,102]

### 5.1. Dcytb Homologs

Dcytb reduces ferric iron and facilitates iron absorption [27], especially under hypoxic conditions [103]. Mammals also express Lcytb, a close homolog of Dcytb that localizes in the lysosome, but has not yet been studied functionally [104]. The *Drosophila* genome has two homolog genes, termed *CG1275* and *no extended memory* (*nemy*). *nemy* was recovered from a genetic screen for learning and memory mutants [91] and could function in memory formation by regulating intravesicular peptidyl alpha-hydroxylating monooxygenase (PHM) activity and the formation of amidated neuropeptides [92]. Because PHM is a copper-dependent enzyme [105] and Dcytb is known to also reduce copper [106,107], the function of *nemy* in learning and memory may therefore be mediated through copper reduction. A recent report showed that *nemy* is strongly induced by hypoxia [108]. Whether *nemy* has a role in iron absorption remains to be elucidated. Likewise, *CG1275* has not been studied to date.

### 5.2. HCP1 Homolog

Soon after the proposal that HCP1 is the heme import protein localized in the apical membrane of the duodenal enterocytes [28], it was shown that the same protein was undoubtedly also (or primarily) serving as a folate transporter [29]. However, further studies have suggested that HCP1 likely transports both heme and folate [109,110,111]. *Drosophila* has a clear homolog encoded by *CG30345*, which has not been studied to date.

### 5.3. FLVCR Homolog

The cellular receptor for feline leukemia virus subgroup C (FLVCR) has been identified as a human heme exporter that is essential for erythropoiesis [112]. Whether FLVCR plays a role in heme absorption from the diet remains unclear [113]; however a clear *Drosophila* homolog (CG1358) for this transporter has been identified and a putative function in the maintenance of circadian rhythms has been ascribed to the fly gene [94].

### 5.4. Heme Oxygenase

*Drosophila* HO has been biochemically characterized [95]. *HO* RNAi led to larval and pupal lethality, with a doubling of heme content measured in the affected individuals [96]. More experiments are required to evaluate if dietary heme represents a source of iron for *Drosophila* and consequently whether HO has a specific function in the intestine during the process of intestinal iron absorption or not. Interestingly, according to Flybase, *HCP1* shows a developmental peak in expression at the end of the third instar larva (after the larvae cease to eat) and of *HO* during the first day of metamorphosis (when extensive histolysis takes place) [93].

### 5.5. HIF

Sima and Tango are the HIFα and HIFβ homologs in *Drosophila* [97]. Several laboratories are using *Drosophila* to study the hypoxic response, however these works are beyond the scope of this review and the reader is referred to reviews of the literature in this rapidly expanding field [98,99,100]. Iron is a cofactor for the prolyl hydroxylase Fatiga and affects Sima stability [101] and hypoxia directly affects iron homeostasis in mammals (see Section 2.2. Regulation of Iron Absorption) and other invertebrates [114,115]. Although it has been established that tracheal cells sense hypoxia and induce terminal branch sprouting [102], the effect of sima and tango on iron homeostasis has not been directly investigated to date. 

## 6. Differences in Iron Homeostasis between Mammals and Insects

Mammals and insects rely on different respiratory organs and differ on how they distribute the oxygen in the whole body: Mammals use lungs and the circulatory system to systemically distribute the oxygen whereas the insects use the tracheal system to distribute the oxygen [116]. In mammals, erythropoiesis occurs in the bone marrow and red blood cells are the carriers of oxygen from the lungs to tissues and of carbon dioxide from the tissues to the lungs. The iron requirement for hemoglobin production by far exceeds all other demands for iron [117]. In contrast, the insect tracheal system is a continuous tubular network that provides air directly to every organ and tissue throughout the body. Therefore, the insect circulatory system, which is open, is not primary used to transport oxygen and *Drosophila* hemoglobin is only expressed locally at the tip of the trachea [118,119,120]. Such a major difference in the organization of the respiratory and circulatory systems of insects and mammals is also reflected in the regulation of systemic iron homeostasis: the whole hemochromatosis-related pathway, including hepcidin, is lacking and also there is no EPO in *Drosophila*. Therefore, *Drosophila* is a poor model for some human conditions (*i.e.*, hemochromatosis or some forms of anemia) but an excellent model to study tissue specific functions for iron, in processes that can be “masked” in the vertebrate models because of the physiologic priority of shuttling iron into erythropoiesis.

Another difference that should be kept in mind is the subcellular localization of ferritin, which in *Drosophila* and most other insects resides within the secretory system (ER and Golgi) and is secreted in the hemolymph in large quantities [2,19,52,121]. Although ferritin clearly serves an iron storage function in *Drosophila* [2,15,74] it remains to be shown whether it also serves as a transporter of iron between cells [52], as has been definitively shown for one of two tick ferritins [122]. Below, we briefly discuss the gaps in our current knowledge arising from the lack of conservation from key players of vertebrate iron homeostasis (Table 3).

**Table 3:** Mammalian iron metabolism proteins with no orthologs in *Drosophila*.

Protein name (mammals)	Key questions arising
Ferroportin	How do insects export iron from cells?
Hepcidin	How do insects signal peripheral iron sufficiency?
Erythropoietin	No erythropoiesis in insects; is there a diffusible signal for systemic hypoxia?
Transferrin Receptor	Is there a functional TsfR in flies? What is the function of Tsf1?
	Is there a ferritin receptor and does ferritin mediate systemic iron transport?

### 6.1. Ferroportin

Ferroportin is the only known export protein for ferrous iron in mammalian cells [123]. There is no ferroportin homolog encoded in the *Drosophila* genome [52]. Therefore the question of how iron can exit an insect cell is of paramount importance, and a key unknown factor in the process of intestinal iron absorption. If an iron export protein exists in insects, it will be of interest to see if such a protein is also conserved in mammals. Ferritin secretion could be one mechanism of cellular iron export [52,122], although it would mean that only large quantities of iron could be released at any given time.

### 6.2. Hepcidin

The closest homologs of the iron-hormone hepcidin in *Drosophila* are the antimicrobial peptides of the defensin type [124,125]. These have been implicated in epithelial homeostasis in the *Drosophila* gut through their effects on intestinal microbiota [126]. They may also have an alternative function in systemic iron homeostasis, but this hypothesis requires experimental testing. In any case, it is highly probable that insects will signal their peripheral iron demands to the intestine, perhaps using circulating ferritin as a direct signal [2,70]. 

### 6.3. Erythropoietin

Unsurprisingly, there is no gene encoding for a protein with similarity to erythropoietin present in the fly genome. Nevertheless, it is worth asking if hypoxia releases any humoral factor in flies; in this sense the recent implication of estrogen-related receptor in the hypoxic response is intriguing [108]. The Malpighian tubules of *Drosophila* are its major excretory organ [127,128]. The Malpighian tubules clearly receive and respond to signals of stress or immune challenge [129], but it is not clear if they in turn secrete any hormones into the hemolymph, or if this is a function reserved for the fat bodies and other glands. 

### 6.4. Transferrin Receptor

There has been a fair amount of discussion in reviews of insect iron metabolism over the fact that no Transferrin Receptor gene was found by homology searches in insect genomes [52,53,55]. Given the abundance of Tsf1 in the hemolymph, it is very likely that an uptake system for this protein exists. Either it has the same ancestral gene as mammalian TsfR, but has diverged so much in sequence that it cannot be recognized as such, or an independent Tsf uptake system exists in flies. Therefore, until functional experiments are performed it is better to leave the question of an insect TsfR open. The situation is somewhat similar with respect to putative ferritin receptors in mammals, where a few proteins have been suggested to participate in serum ferritin binding and internalization, but conclusive functional evidence is largely missing [130,131,132,133,134,135]. If hemolymph ferritin does indeed traffic iron from one cell to another, then an insect ferritin receptor is another important protein that awaits its discovery and functional analysis.

## 7. Functional Requirements of Iron in *Drosophila*

The functional relevance of iron in *Drosophila* biology transcends its key role in the generation of ATP as a cofactor in Krebs cycle and oxidative phosphorylation enzymes. As discussed above the specific requirement of iron binding to melanotransferrin for the formation of septate junctions in the formation of epithelia [67]. Below we discuss other developmental processes already known to depend on iron proteins. We also describe studies implicating iron in the immune and heat shock responses, the involvement of iron proteins in the maintenance of the circadian rhythm and finally the role of iron in neuronal degeneration, a field of study where many *Drosophila* models have been generated (Table 4) [136].

**Table 4:** Higher biological processes influenced by iron availability.

General process	Specific function	References
Development	Epithelial junction formation	[67]
	Spermatogenesis	[131]
	Cell proliferation	[135,136]
	Ventral furrow formation	[77]
Immune Response	Hemolymph ferritin and transferrin respond to infection	[64,78]
	Zygomycosis	[137]
	Wolbachia	[138,139]
	Sindbis viral entry	[140]
Heat Shock Response	Unknown (ferritin and transferrin are heat shock inducible)	[141,142,143]
Behavior	Taste perception	[63,69]
	Circadian Rhythm	[94]
Human Disease Models	Friedreich’s Ataxia	[9,144,145,146,147,148,149,150,151]
	Alzheimer’s and Parkinson’s Disease	[152,153,154,155]
	Restless Legs Syndrome	[156,157]
	Neurodegeneration	[158,159,160,161]

### 7.1. Iron Requirements for the Development of *Drosophila melanogaster*

From the genes studied so far, *Fer1HCH*, *Fer2LCH* and *Tsf2* mutants are embryonic lethal [2,67], whereas *Mvl* and *MCO3* mutants survive to adulthood [70]. *Drosophila* larvae bearing insertions in *Heat shock protein cognate 20*, a gene required for iron-sulfur cluster assembly, failed to grow during the 3rd instar larval stage and never initiated metamorphosis [90]. *Drosophila* mutant larvae for *aminolevulinate synthase*, encoding for the rate-limiting enzyme in heme biosynthesis, suffered massive water loss, possibly due to failed formation of a dityrosine-based cuticular barrier [162]. Mutants in the *mitoferrin* (*mfrn*) gene reached adulthood, but were male-sterile [163]. As mfrn is thought to transport iron into the matrix of mitochondria [164], it appears that mitochondrial iron is essential for spermatogenesis [163,165]. Whether the testis-specific mitochondrial ferritin (*Fer3HCH* [69]) or other iron genes have a role in this process has not yet been demonstrated; though a mutant recovered in a genetic screen for altered iron homeostasis [166] turned out to be male-sterile under iron deficient conditions (T.P. and F.M. unpublished observations). Iron has also been implicated in cell proliferation [167] and ferritin iron has been suggested to serve as a mitogen [168]; therefore one possibility is that the cell proliferation of spermatids requires an adequate supply of iron to occur. In any event, our present understanding of the role of iron in developmental processes remains rather limited, but there is sufficient evidence that disrupting iron homeostasis affects normal embryogenesis [2] and dietary iron chelation halts larval growth [69]. The recent use of the Synchrotron to visualize metals in tissues adds a further high-resolution *in situ* methodology for the study of the mutants already available [169].

### 7.2. Iron and the Immune Response

Iron sequestration is an important and evolutionarily conserved component of the innate immune response, because acquisition of iron is vital for pathogenic growth [170]. The importance of iron in *Drosophila* immunity can be demonstrated by the fact that regulation of iron proteins is increased in the presence of bacterial or fungal infections; specifically expression of *Tsf1* mRNA [64] and protein [78] increases and a specific cleavage in Fer2LCH has also been detected [78]. Given that the mechanisms between the innate immune systems of both *Drosophila* and human are highly conserved [171], *Drosophila* may be used in trying to understand molecular aspects of poorly understood pathobiology in humans. For instance, it is known that individuals with elevated serum iron levels are at increased risk for zygomycosis, indicating a role of iron metabolism in the pathobiology of zygomycosis [172]. When *Drosophila* was injected with a standardized amount of Zygomycetes spores, rapid infection and death of wild type flies was observed, which could be partially blocked by iron chelation [137]. Similarly, *Wolbachia*, a natural symbiotic host of insects, directly affected ferritin levels in its hosts [138,139]. Beyond the “iron wars” between host and bacterial or fungal pathogens, DMT1 was recently identified as the cellular receptor for Sindbis virus, a prototypical member of the mosquito-borne alphaviruses [140]. As with the role of iron in development, it looks like there is much more left to discover over iron’s multiple functions in the provision of immunity.

### 7.3. Iron and the Heat-Shock Response

Three recent studies have shown significant induction of Tsf1 and ferritin proteins in response to growing the flies at high temperatures [141,142,143]. The precise function of these proteins in protection from heat stress remains unclear.

### 7.4. Iron Influences the Behavior of *Drosophila melanogaster*

The first evidence that mutations in a single gene could affect the behavior of an organism was obtained in studies of the circadian rhythms of *Drosophila* [173]. Rhythmic behavior is mediated by a group of about 150 “circadian” neurons in the central brain [174]. Heme has been previously implicated in the function of the circadian clock [175,176] and is a cofactor in relevant *Drosophila* nuclear receptors [177,178]. Our own investigations on whether heme biosynthetic genes were required in “circadian” neurons were inconclusive; instead we discovered that iron-sulfur cluster biosynthesis genes were implicated in the maintenance of circadian rhythms [94]. We also observed phenotypes with RNAi of *Fer2LCH*, but not *Fer1HCH*, and with two other genes: *Tsf3* and *CG1358*, (FLVCR heme exporter homolog) [94]. Overexpression of ferritin in glial cells also led to an age-dependent decline in the ability to sustain circadian rhythms [158]. Support of the notion that iron may be directly involved in the maintenance of circadian rhythms came also from parallel studies in plants, where a retrograde signal from chloroplasts to nucleus signaling an iron deficiency was suggested to affect the period length of the clock [179,180,181,182]. More work is required to elucidate the mechanistic details of how iron may affect the circadian clock in flies. 

Another behavior affected by iron relates to the perception of sweet taste [66,72]. As discussed earlier, Mvl has a key role in the preference for sugar shown by *Drosophila* flies (see Section 4.1). It appears that behavioral attraction to sugar may lie behind honeybee division of labor; addition of different metals in the hives of honeybees changed their foraging behavior [183,184].

### 7.5. Iron and Models of Human Disease

By far the most studied iron-related disease in a *Drosophila* model system is Friedreich’s ataxia [9,144,145,146,147,148,149,150,151], which in humans arises from reduced expression of the iron-sulfur cluster biosynthesis gene *frataxin* [185,186,187]. RNAi of *frataxin* in *Drosophila* resulted in adult flies with locomotion defects [146], likely explained by defective mitochondrial axonal transport and membrane potential [149] but also by the reduced activity of mitochondrial complexes [145]. RNAi of *frataxin* also increased sensitivity to oxidative stress [146,147] and resulted in accumulation of lipids and lipid peroxidation [9]. Iron has also been implicated in the *Drosophila* models of Restless Legs Syndrome [156,157], Parkinson’s [152,153] and Alzheimer’s [154,155] disease and in metal-induced neurodegeneration [159,160,161]. Finally, a number of dietary studies of compounds with iron chelating properties have been reported, but it is difficult to evaluate how much the effects seen in such studies are a direct consequence of a reduction in intestinal iron absorption [188,189,190]. For more information on “*what can Drosophila teach us about iron-accumulation neurodegenerative disorders*” the reader is referred to a recent review [136]. 

## 8. Conclusions

In contrast to other fields of biology, where work in *Drosophila* pioneered our present understanding, iron absorption is understood more extensively in mammals compared to *Drosophila*. This review compared and contrasted the two systems and pointed to future work required. The physiological significance of iron can be ascertained from studies of its impact on neurodegenerative disorders, development, immune response and behavior. Iron accumulation in different *Drosophila* species is conserved [191]. *Drosophila* shares with mammals the following proteins that function in iron absorption: Mvl (DMT1 homolog; iron import), ferritin (iron storage), IRP-1A (iron regulation), MCO1/MCO3 (ferroxidases), Tsf1 and Tsf2 (transferrin and melanotransferrin homologs, respectively). There are other conserved proteins which have not been investigated over a possible role in iron absorption or regulation, including CG1275/nemy (Dcytb homolog), CG30345 (HCP1 homolog), HO and sima/tango (HIF_a/b_ homologs). The differences in physiology between mammals and *Drosophila* may explain why certain key players involved in iron regulation have no known orthologs, like hepcidin, and erythropoietin, whilst the lack of conservation of ferroportin and TsfR are perplexing, since cellular iron uptake and release are fundamental processes that would have been expected to be phylogenetically conserved. Thus, further research in this field is warranted.
